# Topological phase transition and surface states in a non-Abelian charged nodal line photonic crystal

**DOI:** 10.1515/nanoph-2023-0906

**Published:** 2024-02-23

**Authors:** Haedong Park, Alexander Jones, Minkyung Kim, Sang Soon Oh

**Affiliations:** School of Physics and Astronomy, 2112 Cardiff University, Cardiff CF24 3AA, UK; School of Engineering and Physical Sciences, 3120 SUPA, Heriot-Watt University, Edinburgh, EH14 4AS, UK; School of Mechanical Engineering, 65419 Gwangju Institute of Science and Technology (GIST), Gwangju 61005, Republic of Korea

**Keywords:** nodal line, double diamond, photonic crystal, Euler class, non-Abelian topological charge

## Abstract

Topological charges of nodal lines in a multigap system are represented by non-Abelian numbers, and the Euler class, a topological invariant, can be used to explain their topological phase transitions, such as pair-annihilation of nodal lines. Up until now, no discussion of phase transitions of nodal lines in photonic crystals using the Euler class has been reported, despite the fact that the Euler class and topological phase transition have recently been addressed in metallic or acoustic crystals. Here, we show how the deformation of a photonic crystal causes topological phase transitions in the nodal lines, and the Euler class can be used to theoretically predict the nodal lines’ stability based on the non-Abelian topological charge theory. Specifically, by manipulating the separation between the two single diamond structures and the extent of structural distortion, we numerically demonstrate the topological transition of nodal lines, e.g., from nodal lines to nodal rings. We then demonstrate that the range of surface states is strongly influenced by the topological phase transition of nodal lines. Moreover, the Zak phase was used to explain the surface states’ existence.

## 1 Introduction

Condensed matter physics has advanced extensively with the discovery of topological insulators [1], [2], and numerous concepts about single-gap topologies have been applied to photonics [3], [4], [5], [6]. Recently, multigap topologies [7], [8], [9] have been investigated in addition to single-gap topologies. It is evident that multigap topologies need novel descriptions that are distinct from those used for single-gap topologies. Non-Abelian charges can be used to characterize the multigap degeneracies’ topological nature [10], [11], [12]. Moreover, the braiding of one degeneracy around the other can be explained by the sign change of the non-Abelian charges [10], [11], [13], [14], [15], [16]. Alternatively, the topological phase transitions can be explained by the Euler class, an integer-valued multigap topological invariant [11], [12], [14], [15], [16], [17], [18]. One can determine whether or not two nodal lines passing through a patch can be pair-annihilated by looking at its Euler class.

Since the topological charges of line degeneracies in a three-band system can be described by the simple quaternion numbers, this system provides the simplest platform for studying multigap topologies [10], [16], [19], [20]. Importantly, the descriptions of the phase transition, stability of band degeneracies, and non-Abelian topological charges in three-band systems can be transferred to a system with more bands without losing generality [10], [21]. Nodal lines, which are line degeneracies, can take on a wide variety of curved forms that result in many different topologies [22]. Examples of these topologies include nodal rings [23], [24], nodal chains [20], [25], [26], [27], [28], [29], nodal links [19], [20], [27], [28], [29], [30], [31], [32], [33], [34], and nodal knots [32], [33], [35]. Electronic [10], [11], photonic [19], [20], [26], [36] and acoustic crystals [16], [34] have been used to realize multigap topological nodal line systems and associated phase transitions based on the non-Abelian charge theory.

In a variety of photonic platforms, including metallic photonic crystals and metamaterials, the topological phase transitions of nodal lines have been discovered [3], [4], [5], [20]. They are, however, non-dielectric materials and no dielectric photonic crystal has yet shown the topological phenomena. This is mostly because they require a high refractive index more than 3.5 to have nodal lines isolated from the other bands and creating three-dimensional periodic structures with such materials is difficult [19], [26], [36], [37]. Nevertheless, there are fundamental benefits of using dielectric photonic crystals. Above all, a dielectric photonic crystal is scalable, allowing for the observation of a phenomena that has been predicted theoretically at a wide range of wavelengths, from visible to microwave. In fact, a photonic crystal like the dielectric gyroid has been used to make significant discoveries in the field of topological photonics [3].

In this work, we theoretically investigate the nodal lines’ transition nature with the Euler class and demonstrate their surface states using a double diamond dielectric photonic crystal. To show each nodal line has a non-Abelian topological charge, we first assign the quaternion numbers to the nodal lines created in the multigap system with three bands. Next, we predict the phase transitions of nodal lines, i.e. the stability of nodal lines, by numerically calculating the Euler class. Lastly, we observe the structural deformation-induced transition of surface states in the photonic crystal. We use the double diamond unit cells to calculate the Zak phase for selected paths in momentum space and the double diamond structures’ supercells to simulate the surface states. As such, our research and numerical results provide valuable insight and tools for the exploitation of nodal lines in photonic nanostructures.

## 2 Frame rotation charge, Euler class and topological phase transition of nodal lines

**Figure 1:**
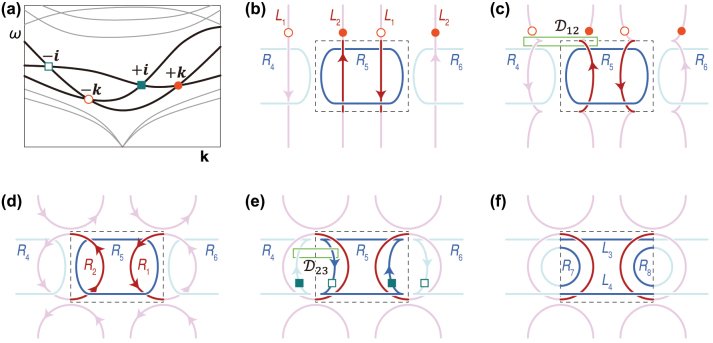
Schematics of phase transitions of non-Abelian charged nodal lines. (a) Quaternion charges of a three-band system in a multiband system. We select the third, fourth, and fifth bands as the member of the three-band system. (b–d) Phase transition of nodal lines *L*_1_ and *L*_2_ into nodal rings *R*_1_ and *R*_2_ to form a nodal link by *R*_4_, *R*_5_ and *R*_6_. (e–f) Phase transition of nodal rings *R*_4_, *R*_5_ and *R*_6_ into nodal rings (*R*_7_ and *R*_8_) and nodal lines (*L*_3_ and *L*_4_). In (b)–(f), red and blue lines are degeneracies by the third-fourth bands and fourth-fifth bands, respectively. The dotted box in each panel means a Brillouin zone.

First, we define the multigap topology. Let us start with a system that consists of (*n* + 1) bands without any degeneracy between them. Then, the system has *n* bandgaps. If a deformation of the band structure makes only one pair of bands degenerate, or if we focus on only one specific pair of bands by ignoring degeneracies formed by any other pairs of bands, the system is called a ‘single-gap system’. Similarly, a ‘multigap system’ is a system where we deal with multiple degeneracies formed by two or more different pairs of bands [14], [16]. For example, some degeneracies are by the first-second bands, while the other degeneracies are by the second-third bands. Thus, the multigap topology is defined as the topology of degeneracies in the multigap system. In this work, we consider photonic band structures with many (more than five) bands but we select only the third, fourth, and fifth bands forming a three-band system as shown in Figure 1(a). Note that the three bands (thick solid lines) are not required to be separated from the rest of the bands (thin grey solid lines) for the discussion of the non-Abelian band topology if we focus on the topological phase transitions of nodal lines instead of looking at the topology of the bands for the whole Brillouin zone.

Nodal lines in a three-band system are characterized by the frame rotation charges expressed as non-Abelian quaternion numbers [10] and their phase transitions are governed by these topological charges. When degeneracies are created by the first-second bands or the second-third bands, their topological charges are denoted as quaternion numbers in the non-Abelian quaternion group $Q=\left\{\pm i,\pm j,\pm k,\pm 1\right\}$ [10]. The degeneracies by the lowest two bands carry the topological charges in the same letter (e.g., + ***k*** or −***k***), and the topological charges’ letter of the degeneracies by the upper two bands then becomes different (e.g., + ***i*** or −***i***). The quaternion numbers ***i***, ***j***, and ***k*** are defined such that ***i*** ^2^ = ***j*** ^2^ = ***k*** ^2^ = −1 [22], leading to the equivalent relations of $\left(+i\right)\left(-i\right)=\left(+j\right)\left(-j\right)=\left(+k\right)\left(-k\right)=+1$ . In the example shown in Figure 1(a), degeneracies by the third-fourth (fourth-fifth) bands counting from the lowest band have the frame rotation charges ± ***k*** (± ***i***) [20]. Although the charges’ signs are gauge-dependent [10], [13], [38], their relative relations remain unchanged unless a braiding is applied [11], [13], [14], [16]. Thus, we can assume that the topological charges of the nodal lines *L*_1_ and *L*_2_ in Figure 1(b) have opposite signs with −***k*** and + ***k***, respectively. The frame rotation charges allow us to determine whether two nodal lines can be annihilated or not. If two degeneracies’ topological charges are respectively + ***k*** and −***k*** so that their compound topological charges are +1, they can be pair-annihilated. In contrast, if these degeneracies have the same topological charges that makes the compound topological charges −1, the degeneracies are considered as stable, i.e., they cannot be pair-annihilated [10], [11], [16].

To predict topological phase transitions of nodal lines, it is more intuitive to use the patch Euler class, which is an integer topological invariant defined for a patch [11], [12], [14], [15], [16], [17], [18]. For eigenstates $\left|u_{k}^{m}\right\rangle$ and $\left|u_{k}^{n}\right\rangle$ of two adjacent bands *m* and *n*, respectively, the Euler class *χ*_*mn*_ is given by the difference between a surface and boundary integrals:

(1) $$\chi_{mn}\left(D\right)=\frac{1}{2\pi }\left[\underset{D}{\int }{Eu}^{mn}dk_{a}dk_{b}-\underset{\partial D}{\oint }A\left(k\right)\cdot dk\right].$$

Here, the surface integral’s integrand Eu^*mn*^ is the Euler form:

(2) $${Eu}^{mn}\left(k\right)=\left\langle \nabla_{k}u_{k}^{m}|\times |\nabla_{k}u_{k}^{n}\right\rangle ,$$

and the boundary integral’s integrand is the Euler connection:

(3) $$A\left(k\right)=\left\langle u_{k}^{m}|\nabla_{k}u_{k}^{n}\right\rangle .$$

The Euler class *χ*_*mn*_ vanishes when there is no nodal lines going through the patch or two nodal lines with quaternion charges with opposite signs go through the patch. This leads to a simple statement that the degeneracies are stable for a non-zero Euler class and unstable for a zero Euler class.

For example, to consider the Euler class, we set a patch $D_{12}$ where *L*_1_ and *L*_2_ pass through (Figure 1(c)). The Euler class calculated over the patch $D_{12}$ is zero due to *L*_1_ and *L*_2_’s opposite charges. This means that the two nodal lines can be pair-annihilated to become nodal rings, as shown in Figure 1(d), and the nodal lines now form a nodal link similar to the one reported in ref. [20]. By focusing on the nodal rings *R*_4_ and *R*_5_, we can apply the same explanation to the nodal lines’ phase transitions. Their charges’ signs (+ ***i*** and −***i***, respectively) are opposite as shown in Figure 1(e). The Euler class calculated over the patch $D_{23}$ is then zero so that *R*_4_, *R*_5_ and *R*_6_ can be pair-annihilated to transform into nodal rings (*R*_7_ and *R*_8_) and nodal lines (*L*_3_ and *L*_4_) (see Figure 1(f)).

## 3 Double diamond photonic crystal for photonic nodal lines

**Figure 2:**
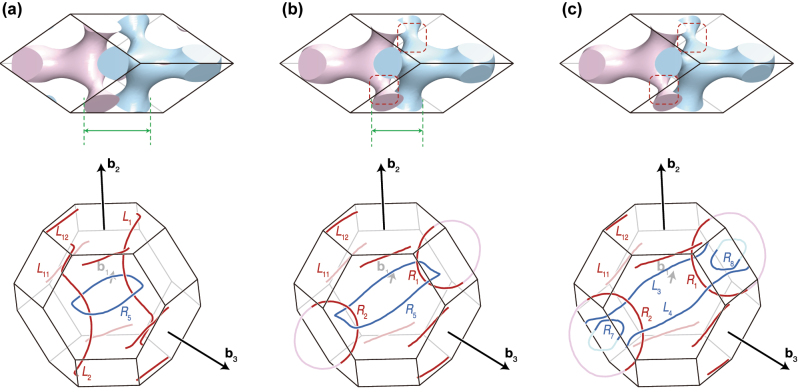
Phase transitions of nodal lines in selected double diamond structures. The top row displays the unit cells in the real space, and the bottom row exhibits their nodal lines in the momentum space. Structural deformations are highlighted as green arrows and red dotted boxes. The distance adjustment between the two single diamonds from (a) to (b) are denoted by the green arrows. Changing the thickness of the arms from (b) to (c) are marked by the red dotted boxes. Except the nodal lines *L*_11_ and *L*_12_, each configuration of nodal lines in (a), (b), and (c) correspond to Figure 1(b), (d), and (f), respectively.

To realize the aforementioned phase transitions, we adopt the dielectric double diamond structure (see Figure 2) introduced in ref. [20], which exhibits the nodal lines in the 3D momentum space. The double diamond consists of two single diamonds that are mutually inversion symmetric with respect to the origin. One single diamond is expressed as the set of $x=\left[x_{1},x_{2},x_{3}\right]$ satisfying $f\left(x\right)>f_{c}>0$ . Here, *f*_*c*_ is the cutoff value that determines the volume of the structure. The triply periodic function $f\left(x\right)$ is expressed as

(4) $$\begin{array}{ll} f\left(x\right) & =A_{0}\sin\left(X_{1}+X_{2}+X_{3}\right)\\ & \;+\overset{3}{\underset{i=1}{\sum }}A_{i}\sin\left(X_{1}+X_{2}+X_{3}-2X_{i}\right) \end{array}$$

where *A*_0_, *A*_1_, *A*_2_, and *A*_3_ determine the detailed shape of the single diamond. The normalized local coordinate **X** is written as $X=\left[X_{1},X_{2},X_{3}\right]=\left(2\pi /a\right)\left(x-\gamma a/2\right)$ where *a* is the lattice constant, $a=\sum_{i=1}^{3}a_{i}$ is the summation of the lattice vectors, and *γ* is the coefficient that tunes the distance between two single diamonds along the **a**-direction. The other single diamond, the counterpart of the above single diamond, is given by the set of **x** such that $f\left(-x\right)>f_{c}>0$ . The sets of **x** satisfying $f\left(x\right)>f_{c}>0$ and $f\left(-x\right)>f_{c}>0$ are displayed as pink and cyan structures, respectively, in the top row of Figure 2. Both the two single diamonds’ dielectric permittivities are 15.0 regardless of their colors.

This representation of the three-dimensional geometry gives us a convenient way to realize PT -symmetry protected nodal lines [3], [13] without redundant degeneracies. Note that the double diamond structure given by Eq. (4) is inversion and time-reversal symmetric for any parameters so that nodal lines instead of Weyl points can be generated, and all the nodal lines in the momentum space is inversion symmetric for the Γ-point. To remove redundant degeneracies such as doubly-, triply-degenerate surfaces, all four *A*_*i*_ are chosen to be different, and *γ* is chosen to be non-zero. These choices prevent having the other spatial symmetries such as rotation and mirror symmetries that can lead to additional degeneracies.

To obtain the nodal lines of the double diamond photonic crystal, we take on the third, fourth, and fifth bands in the numerically calculated photonic band structure and focus on two types of degeneracies, denoted as red and blue lines at the bottom row of Figure 2, created by these three bands (the detailed calculation method is in Supplementary Material, Section S3)

## 4 Demonstration of phase transitions of photonic nodal lines

To demonstrate the phase transitions, we control only the two parameters *γ* and *A*_1_, although the other structural or material parameters are also related to the control of the detailed geometry of the double diamond. *γ* is for tuning the distance between the single diamonds (see the structures and green arrows in Figure 2(a and b)). We change *γ* from 0.02 (Figure 2(a)) to 0.08 (Figure 2(b and c)). *A*_1_ is for adjusting the thickness of each single diamond’s arm (see the structures and red dotted boxes in Figure 2(b and c)). *A*_1_ decreases from 1.19 (Figure 2(a and b)) to 1.05 (Figure 2(c)). The other values remain constant and are summarized in Supplementary Material, Section S3.

Degeneracies by the third-fourth bands and fourth-fifth bands in the band structures are plotted as nodal lines as shown in the bottom row of Figure 2. Commonly they have six nodal lines around their boundaries. By considering the periodicity of the Brillouin zone, the six nodal lines can be classified as two groups according to their connectivity [20], and we denote them as *L*_11_ and *L*_12_, as marked in Figure 2. If we ignore *L*_11_ and *L*_12_, we can regard that Figure 1(b), (d), and (f) are realized as each panel of Figure 2, respectively. By adjusting the distance between the two single diamonds (from Figure 2(a) to (b)), the nodal lines *L*_1_ outside the Brillouin zone and *L*_2_ inside the Brillouin zone exchange their connectivity to form *R*_1_ and *R*_2_. By decreasing the thickness of each diamond’s arm (from Figure 2(b) to (c)), the vertices of the nodal ring *R*_5_ go outward from the Γ point. Each vertex meets the neighbor Brillouin zone’s nodal ring’s vertex (refer to *R*_4_, *R*_5_, and *R*_6_ in Figure 1(e)). Finally, they transform into two nodal rings (*R*_7_ and *R*_8_) and two nodal lines (*L*_3_ and *L*_4_).

**Figure 3:**
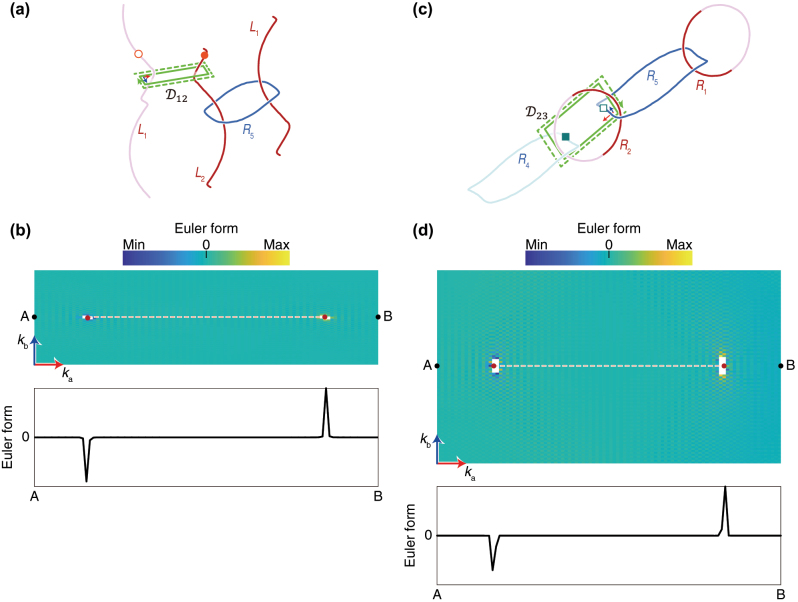
Analysis of phase transitions of nodal lines using the Euler class. (a) Nodal lines of Figure 2(a) and the patch $D_{12}$ to calculate the Euler class. Only *L*_1_, *L*_2_ and *R*_5_ are displayed. (b) 2D distribution of Euler form numerically calculated over $D_{12}$ (upper) and its 1D slice plot on the line that connects the points A and B on the 2D plot (lower). (c) Nodal link of Figure 2(b) and the patch $D_{23}$ to calculate the Euler class. Only *R*_1_, *R*_2_, *R*_4_ and *R*_5_ are displayed. (d) 2D distribution of Euler form numerically calculated over $D_{23}$ (upper) and its 1D slice plot on the line that connects the points *A* and *B* on the 2D plot (lower). In (a) and (c), the green solid line rectangles mean the patches while the green dotted arrow-lines indicate the boundary integral direction of the Euler connection. The patches in (a) and (c) correspond to $D_{12}$ and $D_{23}$ in Figure 1(c) and (e), respectively. In the 2D plots in (b) and (d), the red dots mean the points pierced by the nodal lines, and the dotted lines are the Dirac strings [11], [16].

To see the stability of the photonic nodal lines, the Euler class is calculated over a two-dimensional patch where an even number of nodal lines pass through. Here, the nodal lines are the ones formed by the same pair of bands. As mentioned in Section 2, if the Euler class is zero (nonzero), the nodal lines can be (cannot be) pair-annihilated or pair-generated so that the phase transition is feasible (unfeasible). To analyze the phase transition of *L*_1_ and *L*_2_ in Figure 2(a and b), we set a patch $D_{12}$ pierced by the nodal lines *L*_1_ and *L*_2_, as shown in Figure 3(a). The two nodal lines have oppositely signed frame rotation charges expressed as −***k*** and + ***k***, respectively. The numerically calculated Euler form (see Figure 3(b)) is zero in the overall region except around the nodes where *L*_1_ and *L*_2_ are going through the patch. Around the *L*_1_ and *L*_2_ (the small white-cut regions in Figure 3(b)), the Euler form goes towards the −∞ and +∞, respectively. Thus, the surface integral becomes zero. For the given patch, the boundary integral is zero, too. Therefore, the Euler class calculated over $D_{12}$ becomes zero, and we can conclude that the nodal lines *L*_1_ and *L*_2_ can be pair-annihilated to be transformed into a ring creating a nodal link in the whole Brillouin zone.

The same analysis is carried out for the case in Figure 3(c and d). The numerically calculated Euler class over $D_{23}$ is zero because both surface and boundary integrals are zero. Thus, the oppositely charged nodal lines *R*_4_ and *R*_5_ have the same property as *L*_1_ and *L*_2_ in the previous paragraph, and they can deform into Figure 2(c). Figure 3(b) and (d) plot only real part of the Euler form. The distribution of this quantity’s imaginary part and the relevant discussion are in Supplementary Material, Section S1. The numerical calculation method of the Euler class is also given in Supplementary Material, Section S1.

## 5 Zak phase and surface states

**Figure 4:**
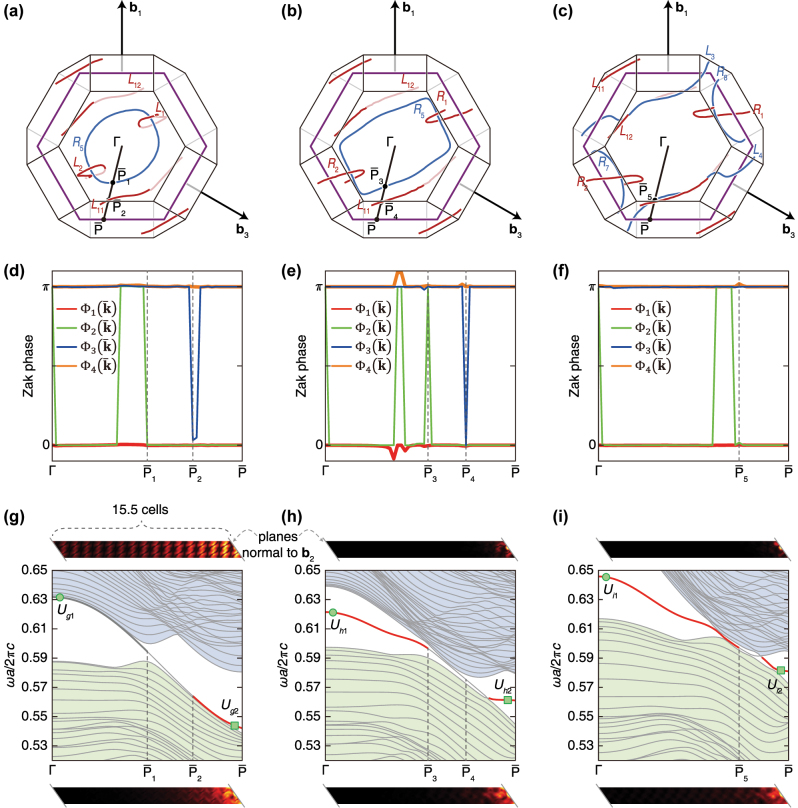
Phase transition of surface states for the three cases in Figure 2. The first, second, and third column correspond to Figure 2(a)–(c), respectively. In each column, all the figures in the first row indicate nodal lines and paths in the momentum space of a unit cell, where they are viewed from **b**_2_. All the hexagons are normal to **b**_2_. All the figures in the second and third row are the Zak phases and band structures, respectively, along the paths $\Gamma \bar{P}$ in the first row. The Zak phases are calculated using the unit cell of each case, while we suppose a supercell that consists of the 15.5 double diamond unit cells to calculate the band structures. The eigenstates at the points marked by the circles and rectangles in each band structure (in the last row) are displayed above and below the band structure, respectively. Another view of (a)–(c) is in Supplementary Material, Section S5.

So far we have shown the topological phase transition of photonic nodal lines and have explained their stability using the Euler class. Now, we will consider how the topological phase transition affects the existence of surface states. In general, surface states are closely related to the bulk bands as they are located inside the full or directional bandgaps. For the case of topological insulators, the surface states should be present due to the non-trivial topological phases of bulks that are described by Chern numbers or Zak phases. This is the well-known bulk-surface correspondence [39], [40]. However, there is no equivalent relation between the surface states and the non-Abelian topological charges of nodal lines. One can instead relate the surface states with the Zak phases of the bulk bands which can then be related to the phase transition of nodal lines. In this section, we first establish the link between the Zak phase [41] and the Wilczek-Zee phase [42] of nodal lines and then show the numerical results of the surface states and Zak phases calculated using 4 × 4 Wilson loop. Here, to create a surface of the double-diamond structure, we select the plane normal to **b**_2_ (the hexagon in Figure 4(a)–(c)) because this plane is almost parallel to the nodal ring *R*_5_ so that we can easily observe surface states.

**Figure 5:**
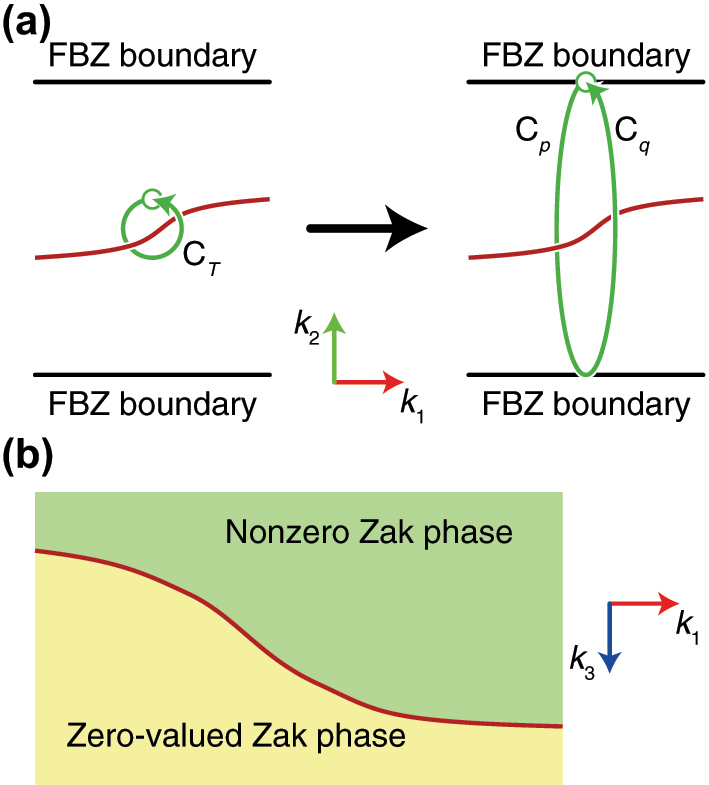
Schematics to explain the Zak phase. (a) A nodal line (the red curve) and a closed loop (the green curve) being stretched from *C*_*T*_ along the *k*_2_-direction so that it touches the boundaries of the first Brillouin zone. The two boundary-to-boundary curves are denoted as *C*_*p*_ and *C*_*q*_, i.e., *C*_*T*_ = *C*_*p*_ + *C*_*q*_. (b) The nodal line projected onto the plane normal to *k*_3_-direction.

The Zak phase is calculated by integrating the Wilczek-Zee connection along a boundary-to-boundary straight line. The start and end points of the integration path are equivalent as they are on the Brillouin zone boundaries. Meanwhile, the topological charge of a nodal line can also be calculated by integrating the Wilczek-Zee connection [22], [42] along a closed loop that encircles a point of the nodal line. Then, let us assume a nodal line partially or fully parallel to the boundary and a small elliptic closed loop (Paths *C*_*T*_ in Figure 5(a)). If we stretch the closed loop such that its ends respectively touch the boundaries, two boundary-to-boundary curves (Paths *C*_*p*_ and *C*_*q*_ in Figure 5(a), respectively) can be approximated as the straight paths used for the Zak phase calculation. The integral of the Wilczek-Zee connection along the closed loop *C*_*T*_ is ±π. Then, among the two integrals of the Wilczek-Zee connection along *C*_*p*_ and *C*_*q*_, only one is ±π while the other is zero, because the eigenstates at the two ends of *C*_*p*_ (or *C*_*q*_) are equivalent (Note that the inner product of the eigenstates at the two ends is +1 or −1). In other words, among the two Zak phases along the two paths, only one becomes nonzero. Thus, the nodal line viewed from the normal vector of the boundaries acts as the border curve that switches the Zak phase between zero and nonzero, as shown in Figure 5(b), if surrounding bands do not generate another degeneracies with the band involved in the aforementioned nodal line around the location of the nodal line.

We calculate the Zak phase for the three different double diamond unit cells in Figure 2. First, the same $\Gamma \bar{P}$ lines are prepared for each Brillouin zone (see Figure 4(a–c)), where $\bar{P}$ is placed on the edge of the surface hexagon. At each point $\bar{k}$ on $\Gamma \bar{P}$ , we calculate the Zak phase $\Phi_{i}\left(\bar{k}\right)$ whose integral is performed along the line that starts at $\bar{k}-b_{2}/2$ , passes $\bar{k}$ , and finishes at $\bar{k}+b_{2}/2$ . The eigenstates $\left|u_{k}^{p}\right\rangle$ (*p* = 1, 2, 3, 4) are used to build the 4 × 4 Wilson loop $W_{pq}$ . The Wilson loop’s eigenvalues’ arguments become the Zak phases Φ_*i*_ (see Supplementary Material, Section S2) [37], [43], thus we do not know which bands are related to each Φ_*i*_. Meanwhile, the reason of constructing the Wilson loop in 4 × 4 form is that we want to see what happens between the fourth and fifth bands. In other words, as the directional bandgap is generated between the fourth and fifth bands, we gather the band information below the bandgap.

Therefore, instead of obtaining much information about surface states from the Zak phase, we observe the Zak phases’ evolution with deformation of the double diamond unit cell. Among the four Zak phases, Φ_1_ and Φ_4_ are always zero and π, respectively. The remaining eigenvalues Φ_2_ and Φ_3_ are quantized as zero or π, (as shown in Figure 4(d–f)). Points ${\bar{P}}_{i}$ are the intersections of $\Gamma \bar{P}$ and the projection of nodal lines onto the surface hexagon plane. During the deformation of the double diamond from Figure 4(a) to (b), Φ_2_ that were π around ${\bar{P}}_{1}$ in Figure 4(d) become zero. These go back to π when the phase transition of the nodal lines occurs from Figure 4(e) to (f). The Φ_2_ switches the value around ${\bar{P}}_{1}$ , ${\bar{P}}_{3}$ , and ${\bar{P}}_{5}$ , and Φ_3_ temporarily goes to zero and gets back to π around ${\bar{P}}_{2}$ and ${\bar{P}}_{4}$ . Thus, we think that these points act as the phase boundaries.

We then simulate the band structures of the double diamond using supercells. The supercells used in Figure 4(g–i) consist of 15.5 cells of the unit cells in Figure 2(a–c), respectively. Each band structure is divided into three regions: (1) the longest region that starts from the Γ point, (2) the region that heads to the point $\bar{P}$ , and (3) the intermediate region. Although there are directional-bandgaps in the first and last regions in all the three cases, the eigenstates behaviors between these three cases are a little bit different. The eigenstate *U*_*g*1_ in Figure 4(g) does not clearly exhibit the surface localization, while the photonic wave is in a localized *U*_*g*2_ state. Another four states marked in Figure 4(h and i) display the localization of photonic waves. We also observe the decrease of the length of the intermediate region from Figure 4(g) to (i). This can be understood as clarifying the surface band isolation, and it corresponds to the degree of localization of photonic waves.

However, the trend of such Zak phases do not completely coincide to the location of surface states of the supercells. This is due to the other bands’ degeneracies. For example, if there is a degeneracy between the first and second bands, the Zak phase may be affected. Further discussion is provided in Supplementary Material, Section S2.

**Figure 6:**
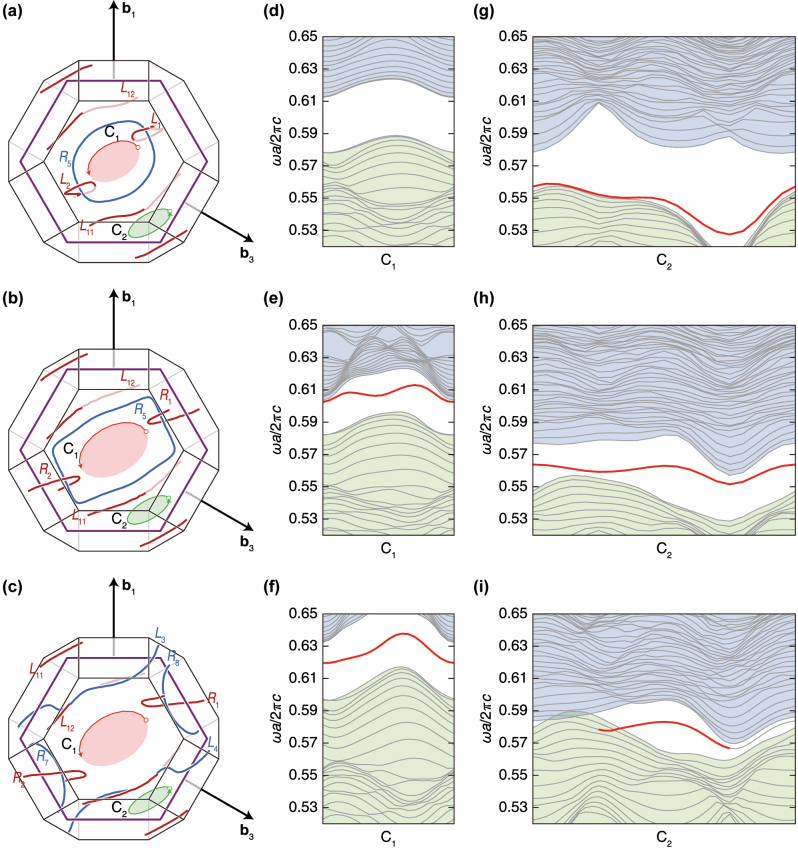
Observation of surface states for the selected area. The situations of each row are equivalent to each column of Figures 2 and 4. The nodal lines and paths in the momentum space of a unit cell are shown in the first column of each row. The band structures along the curved paths 1 and 2 marked in the first column are displayed in the second and third column of each row. Another view of (a)–(c) is in Supplementary Material, Section S5.

To elaborate the relation between the nodal line topology and surface states, we show more projected band structures for several additional paths. We put two paths onto each hexagon as shown in Figure 6(a–c) where the nodal lines are by Figure 2(a–c), respectively. One is a half ellipse (the curve *C*_1_), and the other one is a loop (the curve *C*_2_), as shown in Figure 6(a–c). Although the curves’ exact locations and shapes changes slightly, their relations with the surrounding nodal lines are maintained from Figure 6(a) to (c), meaning that the paths do not cross any nodal lines in the projected band structures. In our band calculations, we keep the same frequency range as the one for Figure 4(g–i).

The band structure along the curve *C*_1_ in Figure 6(d) does not exhibit a sufficiently isolated band related to the surface states. With deforming the double diamond from Figure 2(a) to (b), a band starts to be isolated, as shown in Figure 6(e). This band becomes more isolated (see Figure 6(f)) when the structure in Figure 2(b) becomes Figure 2(c). The band structures along the curve *C*_2_ show the different behaviors. There is a partially isolated band in Figure 6(g), and its degree of isolation increases in Figure 6(h). However, the length of path that correspond to isolated band decreases in Figure 6(i), i.e., some part of the band that was isolated in Figure 6(h) is now penetrated in the surrounding bulk bands. The calculation details of all the surface states in Figures 4 and 6 are in Supplementary Material, Section S4.

## 6 Conclusions

In summary, we were able to describe the stability of photonic nodal lines in the multigap system by using the Euler class. By tuning the deformation of the double diamond photonic crystal, we have shown the phase transitions of photonic nodal lines in a dielectric photonic crystal transforming from nodal lines to nodal rings. The zero or nonzero-valued Euler class is closely related with the non-Abelian topological charges of the band degeneracies, but it is gauge independent. Thus, the Euler class is a topological invariant that can readily tell us about the stability of the band degeneracies clearly without fixing the gauge of the eigenstates, i.e., the electromagnetic field vectors.

Additionally, the evolution of surface states has been investigated. We have attempted to predict the existence of the surface states with the Zak phase obtained by the Wilson loop calculation. Then, we observed the transition of surface states by calculating the projected band structures for the double diamond supercells. Although we were able to observe some correspondences between the Zak phase and surface states for some selected paths in the momentum space, the bulk-surface correspondence regarding the multigap topology is still in veil. However, we believe follow-up study with optimized parameters for dielectric photonic crystals will give us better understanding and insight.

Finally, it is worth investigating the critical point where the phase transition occurs. In our calculation results, although the results correspond to deformation parameters for before and after the phase transition, we did not show the case for the exact parameters for which the phase transition occurs because finding the structural condition numerically is challenging. Nevertheless, we expect that the nodal line shape for the critical parameter will be a simple nodal chain formed by two nodal lines, although we do not exclude the possibility of more complex form of nodal chains.

## Supplementary Material

Supplementary Material Details
