# Supplementary material for: Topological phase transition and surface states in a non-Abelian charged nodal line photonic crystal
Source: Nanophotonics. 2024 Feb 23;13(7):1079–89. doi: 10.1515/nanoph-2023-0906 (PMC11501625; doi:10.1515/nanoph-2023-0906)
Supplement: Supplementary file 1 — Supplementary Material Details [file j_nanoph-2023-0906_suppl_001.pdf]

Supplementary Material for

"Topological phase transition and surface states in a non-Abelian charged nodal line photonic crystal"

Haedong Park[1], Alexander Jones[2], Minkyung Kim[3], and Sang Soon Oh[1*]

[1]School of Physics and Astronomy, Cardiff University, Cardiff CF24 3AA, United Kingdom

[2]School of Engineering and Physical Sciences, SUPA, Heriot-Watt University, Edinburgh, EH14 4AS, United Kingdom

[3]School of Mechanical Engineering, Gwangju Institute of Science and Technology (GIST), Gwangju 61005, Republic of Korea

*Email: ohs2@cardiff.ac.uk

Contents

1. Euler class to determine nodal lines' stability/instability ------------------ 3

2. Discussion on the Zak phase ------------------------------------ 6

3. Simulation of nodal lines --------------------------------------- 9

4. Surface states calculation details --------------------------------- 10

5. Another view of the first Brillouin zone ---------------------------- 10

1. Euler class to determine nodal lines' stability/instability

Calculation method

The Euler class can be utilized to predict the stability of pair-annihilation of nodal lines. The Euler class is calculated using mathematical formulae, over the plane $\mathcal{D}$ and along the boundary of $\mathcal{D}$. If the plane $\mathcal{D}$ does not have any band node, this Euler class becomes zero because the surface and boundary integrals are cancelled out by Stokes' theorem. If the plane $\mathcal{D}$ has two band nodes, the result can be zero or non-zero. For the oppositely charged nodal lines, the result becomes zero. But if the nodal lines have the same charges, the result becomes one. The zero- and non-zero valued Euler classes mean that the nodal lines can be and cannot be merged, respectively.

We calculated the Euler class by Appendix D.4. in Ref. [1] (that refers Ref. [2]). As an eigenstate of a photonic band structure is complex-valued, some formulae in Eqs (1)-(3) or in Ref. [2] were replaced as follows.

The Euler form is given by the integral over a volume $\mathcal{V}$ in the real space.

$$\mathrm{Eu} = \int_{\mathcal{V}} \left(\frac{\mathbf{U}_{P23} - \mathbf{U}_{P14}}{dk_a}\right)^* \cdot \frac{\mathbf{V}_{P34} - \mathbf{V}_{P12}}{dk_b} - \left(\frac{\mathbf{U}_{P34} - \mathbf{U}_{P12}}{dk_b}\right)^* \cdot \frac{\mathbf{V}_{P23} - \mathbf{V}_{P14}}{dk_a} d\mathcal{V}\,. \qquad (1)$$

where $\mathbf{U}$ and $\mathbf{V}$ mean the eigenstates $|u_{\mathbf{k}}^m\rangle$ and $|u_{\mathbf{k}}^n\rangle$, respectively. The boundary integral of the Euler connection is expressed as

$$\oint_{\partial\mathcal{D}} \langle u_{\mathbf{k}}^m | \nabla_{\mathbf{k}} u_{\mathbf{k}}^n \rangle \cdot d\mathbf{k} = \oint_{\partial D} \left(\int_{\mathcal{V}} \mathbf{U}^* \cdot \frac{\partial \mathbf{V}}{\partial k} d\mathcal{V} \right) \partial k\,. \qquad (2)$$

Finally, the Euler class χ is given by the difference between Equation (1) and (2):

$$\chi = \frac{1}{2\pi}\left[\int_{\mathcal{D}} \mathrm{Eu}\, dk_a dk_b - \oint_{\partial D} \left(\int_{\mathcal{V}} \mathbf{U}^* \cdot \frac{\partial \mathbf{V}}{\partial k} d\mathcal{V} \right) \partial k \right]. \qquad (3)$$

In Figure 3 in the main text, the four vertices of the domain $\mathcal{D}_{12}$ are $-0.6470\mathbf{b}_1 - 0.1334\mathbf{b}_2 - 0.7665\mathbf{b}_3$, $-0.2598\mathbf{b}_1 + 0.1488\mathbf{b}_2 - 0.2789\mathbf{b}_3$, $-0.3530\mathbf{b}_1 + 0.1334\mathbf{b}_2 -$

$0.2335\mathbf{b}_3$, and $-0.7402\mathbf{b}_1 - 0.1488\mathbf{b}_2 - 0.7211\mathbf{b}_3$. The domain $\mathcal{D}_{12}$'s vertices are given by $-0.1476\mathbf{b}_1 - 0.3485\mathbf{b}_2 - 0.2731\mathbf{b}_3$, $-0.6096\mathbf{b}_1 - 0.8325\mathbf{b}_2 - 0.6925\mathbf{b}_3$, $-0.7\mathbf{b}_1 - 0.6795\mathbf{b}_2 - 0.8323\mathbf{b}_3$, and $-0.2380\mathbf{b}_1 - 0.1955\mathbf{b}_2 - 0.4129\mathbf{b}_3$.

There are small white-cut regions around the points pierced by nodal lines in Figure 3(b) and (d). The Euler form on these regions were excluded from the surface integral in Eq. (3).

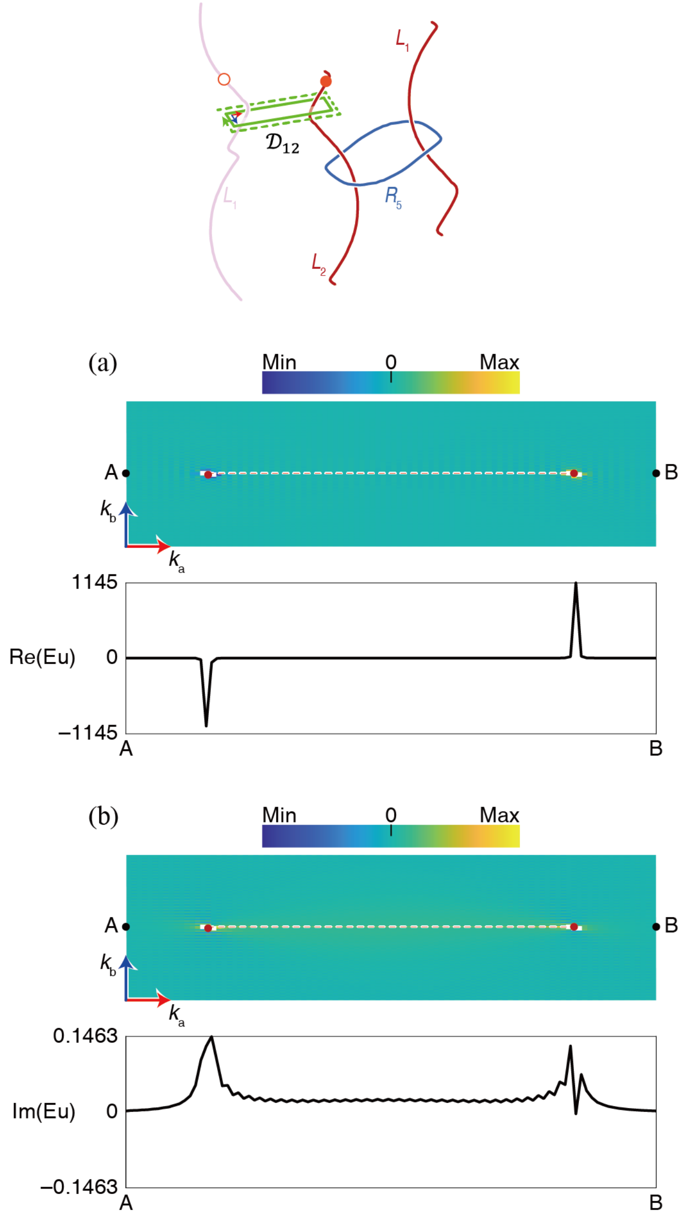

Fig. S1. Plot of the Euler form over $\mathcal{D}_{12}$. The real and imaginary parts of the Euler form Eu are plotted in (**a**) and (**b**), respectively. The two plots in (**a**) are equivalent to Fig. 3(b) in the main text.

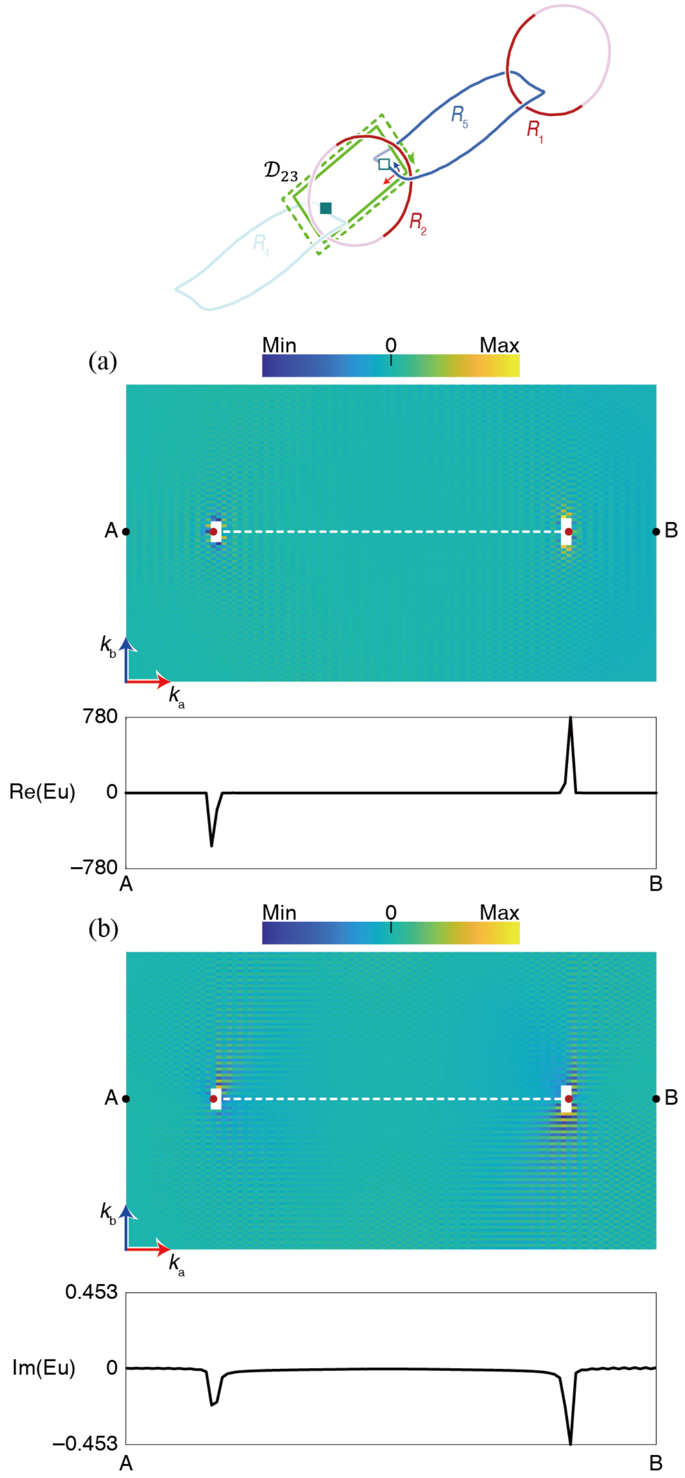

Fig. S2. Plot of the Euler form over $\mathcal{D}_{23}$. The real and imaginary parts of the Euler form Eu are plotted in (**a**) and (**b**), respectively. The two plots in (**a**) are equivalent to Fig. 3(d) in the main text.

Imaginary part of the Euler form

Figure 3 in the main text plots only real part of the Euler form. Compared to the case of using real-valued eigenstates[1-3], we need to compare the real and imaginary parts of the Euler form.

As shown in **Fig. S1** and **Fig. S2**, the maximum of the Euler form's imaginary part's magnitude is negligible compared to the real part. Thus, we determine that there is no issue on the Euler form due to a complex valued eigenstate.

2. Discussion on the Zak phase

Calculation method of the Zak phase

For a given the path $\Gamma\overline{\mathrm{P}}$, we calculated the Wilson loop $\mathcal{W}(\bar{\mathbf{k}})$ in a 4×4 matrix form, i.e., its $[p, q]$-component is given by $\mathcal{W}_{pq}(\bar{\mathbf{k}}) = \exp\left[i \int_C \langle u_{\tilde{\mathbf{k}}}^q | \nabla_{\tilde{\mathbf{k}}} | u_{\tilde{\mathbf{k}}}^p \rangle \cdot d\tilde{\mathbf{k}}\right]$, where p and q are band indices. The integral path C spans from $\bar{\mathbf{k}} - 0.5\mathbf{b}_2$ to $\bar{\mathbf{k}} + 0.5\mathbf{b}_2$, as shown with the green line in **Fig. S3**. If the three eigenvalues of the Wilson loop $[v_1, v_2, v_3]$ are expressed as $[\exp(i\Phi_1), \exp(i\Phi_2), \exp(i\Phi_3)]$, each one's argument $\Phi_i = \mathrm{Arg}(v_i)$ becomes the Zak phases.

Understanding of the Zak phase

The Zak phase distributions along the path $\Gamma\overline{\mathrm{P}}$ (in Figure 4 in the main text) do not exactly coincide to the locations of surface states calculated for the supercells. To address the details, we calculate the 'partial Zak phase'. Let us suppose that, to calculate the Wilson loop, we now perform the line integral from $\mathrm{P_A}$ to not $\mathrm{P_B}$ but only $\tilde{\mathbf{k}}$ a point on the path $\mathrm{P_A P_B}$, i.e., $\mathcal{W}_{pq}(\bar{\mathbf{k}}) = \exp\left[i \int_{\mathrm{P}_A}^{\tilde{\mathbf{k}}} \langle u_{\tilde{\mathbf{k}}}^q | \nabla_{\tilde{\mathbf{k}}} | u_{\tilde{\mathbf{k}}}^p \rangle \cdot d\tilde{\mathbf{k}}\right]$ $(p, q = 1,2,3,4)$. From its eigenvalues, we get their

phases $\Phi_i(\tilde{\mathbf{k}})$ as the partial Zak phase. Thus, the notation $\Phi_i(\tilde{\mathbf{k}})$ means that the path integral during calculation was performed from $\mathrm{P_A}$ only until $\tilde{\mathbf{k}}$.

The plots of partial Zak phases exhibit the sharp switching between zero and π, instead of smooth varying, as shown in **Fig. S4**(a-c). The switching regions coincide to the degeneracy locations. All the three band structures in **Fig. S4**(d-e) have the degeneracies between the first and second bands around the midpoint between $\mathrm{P_A}$ and $\mathrm{P_B}$. The frequency difference between the second and third bands around the first Brillouin zone's boundary in each panel is also very small so that we can predict degeneracies around $\mathrm{P_A}$ and $\mathrm{P_B}$. As the eigenstates' directions sharply change around a degeneracy, $\Phi_i(\tilde{\mathbf{k}})$ switches its value due to the degeneracies.

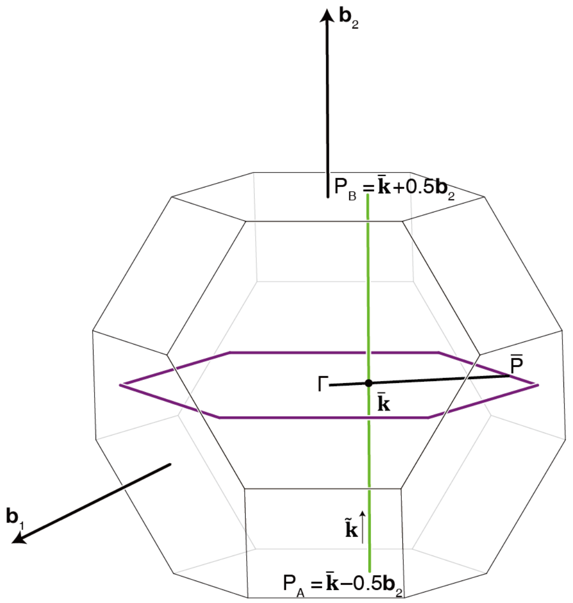

Fig. S3. Example of the path C to calculate the Wilson loop. Here, $\bar{\mathbf{k}}$ and $\tilde{\mathbf{k}}$ indicate points on the paths $\Gamma\bar{\mathrm{P}}$ and $\mathrm{P_A P_B}$, respectively.

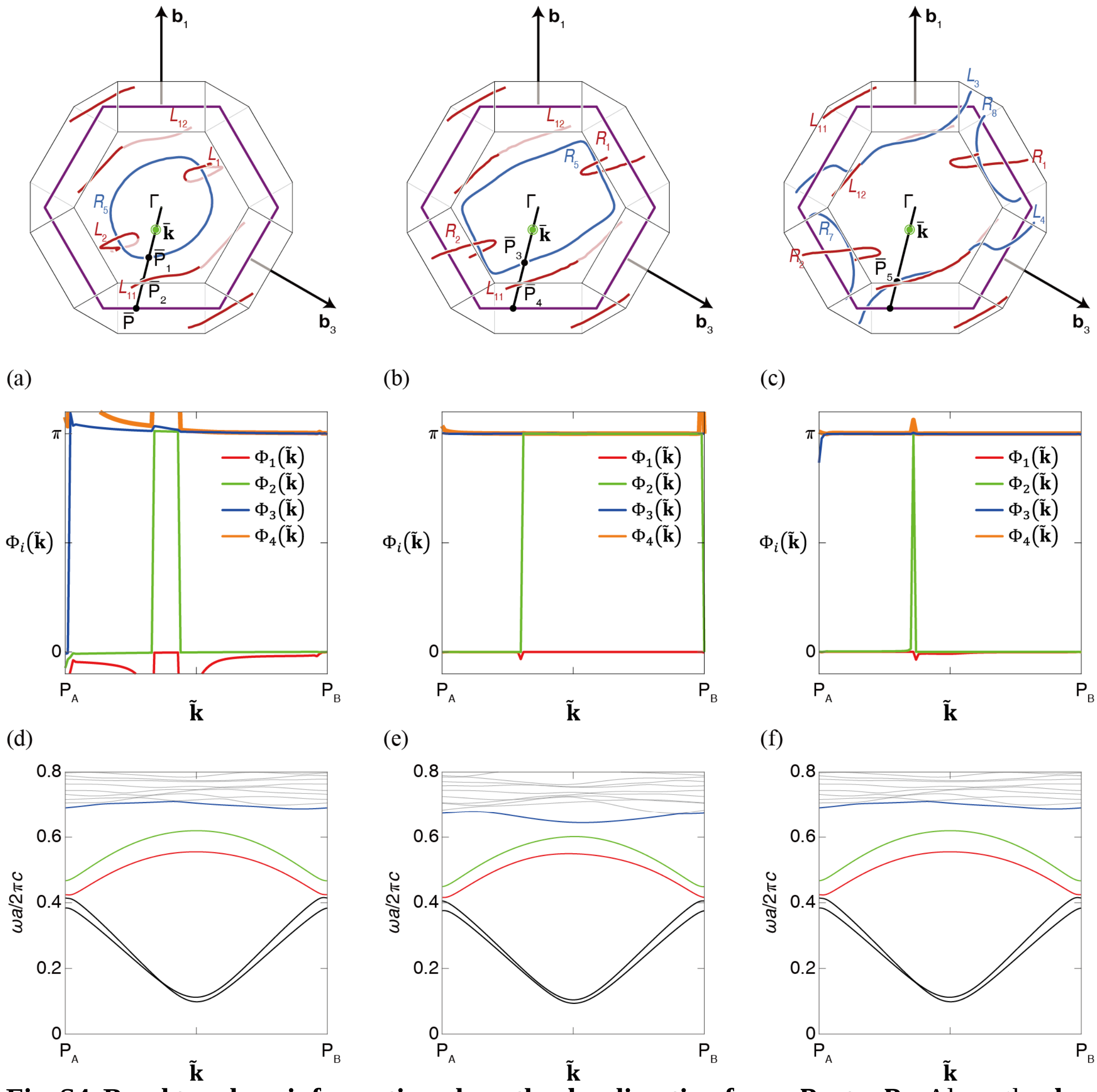

Fig. S4. Band topology information along the $\mathbf{b}_2$-direction from $\mathrm{P_A}$ to $\mathrm{P_B}$. Along the $\mathbf{b}_2$-direction, the path $\mathrm{P_A P_B}$ commonly passes $\bar{\mathbf{k}}$ marked as green circles in the three panels of the first row. **a-c**, Partial Zak phases $\Phi_i(\tilde{\mathbf{k}})$ for $\tilde{\mathbf{k}}$ for the three situations in each panel of the first row. **d-f**, Band structures along the path $\mathrm{P_A P_B}$ in each panel of the first row. The locations where $\Phi_i(\tilde{\mathbf{k}})$ switches in (**a-c**) coincide to the band degeneracies between the first-second bands, the black curves in (**d-f**).

3. Simulation of nodal lines

Unless there is a mention, the structural parameters of the double diamond are as follows: $A_0 = 1$, $A_1 = 1.19$, $A_2 = 1.37$, $A_2 = 1.28$, $\gamma = 0.08$, and $f_c = 1.85$. The electric permittivity and magnetic permeability are always 15.0 and 1.0, respectively.

We obtained nodal lines in the momentum space by the following steps: First, we supposed a face-centered cubic (FCC) primitive cell in the real space, as shown in Figure 2 in the main text, and it was discretized as 16×16×16 grids. For a lattice constant a, the lattice vectors are given by $\mathbf{a}_1 = a[0,1,1]$, $\mathbf{a}_2 = a[1,0,1]$, and $\mathbf{a}_3 = a[1,1,0]$. In the momentum space, we prepared 101×101×51 grids in a cuboid with $-2\pi/a \le k_x \le 2\pi/a$, $-2\pi/a \le k_y \le 2\pi/a$, and $0 \le k_x \le 2\pi/a$. We then calculated the eigenfrequencies ω_n (where n is a band index) at these grid points in the momentum space, using the MIT Photonic-Bands (MPB) package[4]. The results were copied to fill the opposite cuboid with $-2\pi/a \le k_x \le 2\pi/a$, $-2\pi/a \le k_y \le 2\pi/a$, and $-2\pi/a \le k_x \le 0$ according to this system's inversion symmetric nature.

Next, the exact locations of the nodal lines were obtained using the gradient descent method. We built a function where $f_{mn}(\mathbf{k}) = \omega_m(\mathbf{k}) - \omega_n(\mathbf{k})$ $(n = m + 1)$, meaning the difference between two adjacent bands. In the momentum space, searching a degenerate point is equivalent to exploring a location where $f_{mn}(\mathbf{k})$ becomes minimum. We set an arbitrary point $\mathbf{k}_0$ around a nodal line. By calculating $\mathbf{k}_{i+1} = \mathbf{k}_i - \zeta \nabla f_{mn}$ until $|\nabla f_{mn}|$ becomes sufficiently small, we found $\mathbf{K}_j$ a point on a nodal line. Searching the next point on the nodal line also starts by setting another $\mathbf{k}_0 = \mathbf{K}_j + \delta \mathbf{l} = \mathbf{K}_j + \eta(\mathbf{K}_j - \mathbf{K}_{j-1})$. Here, the rate parameters ζ and η are properly selected.

4. Surface states calculation details

The band structures and their eigenstates of a supercell was calculated using the 'Eigenfrequency' solver of COMSOL Multiphysics®. We prepared the supercell which is the stack of 15.5 FCC unit cells along the $\mathbf{a}_2$-direction. The supercell's non-periodic boundaries are normal to $\mathbf{b}_2$-direction. The Perfectly electric conductor was imposed on these boundaries. Other boundaries were set as periodic, by the Floquet periodic boundary condition.

5. Another view of the first Brillouin zone

Figures 4a-c and 6a-c in the main text illustrate nodal lines and paths in the first Brillouin zone viewed from $+\mathbf{b}_2$. Here, we provide another view of them, as shown in **Fig. S5**.

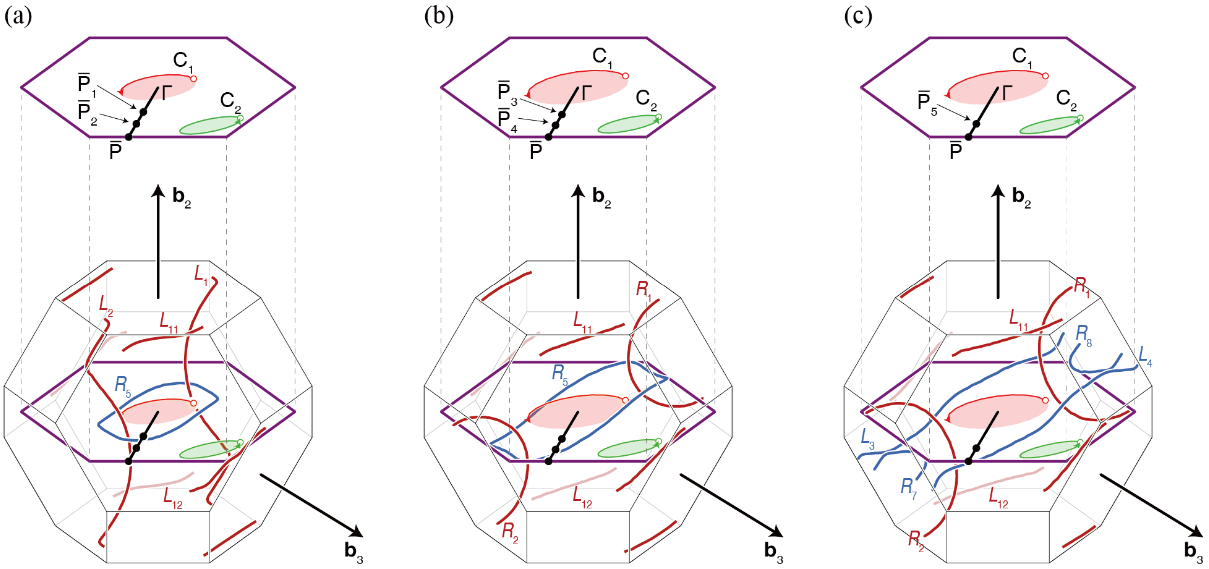

Fig. S5. Another view of Fig. 4(a-c) and 6(a-c) in the main text. The letters of each panel coincide to them of Fig. 4 and 6 in the main text. All the straight and curved paths are on the hexagon.

References

1 Park, H., Wong, S., Bouhon, A., Slager, R.-J. & Oh, S. S. Topological phase transitions of non-Abelian charged nodal lines in spring-mass systems. *Physical Review B* **105**, 214108 (2022).

2 Bouhon, A. et al. Non-Abelian reciprocal braiding of Weyl points and its manifestation in ZrTe. *Nature Physics* **16**, 1137-1143 (2020).

3 Jiang, B. et al. Experimental observation of non-Abelian topological acoustic semimetals and their phase transitions. *Nature Physics* (2021).

4 Johnson, S. G. & Joannopoulos, J. D. Block-iterative frequency-domain methods for Maxwell's equations in a planewave basis. *Opt. Express* **8**, 173-190 (2001).
